# Letter to the Editor regarding the article “Brazilian Academy of Paediatric Otorhinolaryngology Task Force – lingual frenulum disorders in childhood – evidence-based recommendations”

**DOI:** 10.1016/j.bjorl.2026.101824

**Published:** 2026-05-19

**Authors:** Roberta Lopes de Castro Martinelli, Giédre Berretin-Felix

**Affiliations:** a Hospital Santa Therezinha, Brotas, SP, Brazil; b Universidade de São Paulo, Faculdade de Odontologia de Bauru, Department of Speech-Language Pathology and Audiology, Bauru, SP, Brazil

Dear Editor,

We read with great interest the article “Brazilian Academy of Paediatric Otorhinolaryngology Task Force – lingual frenulum disorders in childhood – evidence-based recommendations”.^1^ Given the relevance of this document for clinical practice, we would like to present some methodological observations that may contribute to a more accurate interpretation of the cited literature.

In the section “What are the methods for evaluating the lingual frenulum in children?”, the authors state that “none of the methods for evaluating the lingual frenulum described in the literature have undergone internal or external validation studies”. However, this statement does not appear to reflect the currently available evidence. In particular, reference 17^2^ cited to support this assertion does not correspond to the validation studies of the Lingual Frenulum Protocol for Infants (LFPI)^3^ and its Neonatal Tongue Screening Test (NTST).^4^ This inconsistency creates a traceability issue, since readers cannot identify, from the cited reference, the studies that effectively investigated the measurement properties of these instruments.

The LFPI underwent a structured validation process,^3^ which assessed content, construct, and criterion validity, as well as inter- and intra-examiner reliability, specificity, and predictive values. Similarly, the NTST^4^ was formally validated and demonstrated adequate validity and reliability for use in newborns. Methodologically, these evaluations are consistent with internationally recognized standards such as COSMIN.^5^, ^6^, ^7^, ^8^, ^9^, ^10^

More recently, a systematic review^11^ identified the LFPI as the most comprehensive instrument currently available for the diagnosis of ankyloglossia in infants up to 12-months of age. The interpretation that no lingual frenulum assessment instrument has undergone validation studies may have important clinical and scientific implications, including reduced visibility of published evidence, impaired standardization of assessment, limited comparability between studies, and potential effects on diagnostic and management decisions. We also note that the requirement for professional training in applying the LFPI/NTST should not be interpreted as a weakness. In healthcare, training is inherent to clinical practice. As with history taking and physical examination,^12^, ^13^, ^14^ instruments involving anatomical and functional criteria require standardized observation, specific maneuvers, and appropriate interpretation to ensure diagnostic accuracy and reproducibility.

Finally, the use of the NTST image in the publication raises concerns regarding publication ethics and copyright, as outlined by the WAME.^15^ In light of these considerations, we believe that acknowledging the validation studies of the LFPI and NTST is essential for scientific accuracy, methodological transparency, and the appropriate use of available evidence in clinical practice and public health decision-making.

## ORCID ID

Roberta Lopes de Castro Martinelli: 0000-0002-5791-2575

Giédre Berretin-Felix: 0000-0002-8614-2805

## Data availability

The authors declare that all data are available in repository.

## Conflicts of interest

RLCM and GBF declare a potential conflict of interest, as they are the authors of the tests cited in the manuscript.
